# Transitions From Heart Disease to Cancer as the Leading Cause of Death in US States, 1999–2016

**DOI:** 10.5888/pcd15.180151

**Published:** 2018-12-13

**Authors:** Michael C. Harding, Chantel D. Sloan, Ray M. Merrill, Tiffany M. Harding, Brian J. Thacker, Evan L. Thacker

**Affiliations:** 1 F. Edward Hébert School of Medicine, Uniformed Services University of the Health Sciences, Bethesda, Maryland; 2 Department of Public Health, Brigham Young University, Provo, Utah; 3 Arizona College of Osteopathic Medicine, Midwestern University, Glendale, Arizona

## Abstract

**Introduction:**

Heart disease has been the leading cause of death in the United States since 1910 and cancer the second leading cause of death since 1933. However, cancer emerged recently as the leading cause of death in many US states. The objective of this study was to provide an in-depth analysis of age-standardized annual state-specific mortality rates for heart disease and cancer.

**Methods:**

We used population-based mortality data from 1999 through 2016 to compare 2 underlying cause-of-death categories: diseases of heart (*International Classification of Diseases, 10th Revision* [ICD-10] codes I00–I09, I11, I13, and I20–I51) and malignant neoplasms (ICD-10 codes C00–C97). We calculated age-standardized annual state-specific mortality rate ratios (MRRs) as heart disease mortality rate divided by cancer mortality rate.

**Results:**

In 1999, age-standardized heart disease mortality exceeded that for cancer in all 50 states. Median state-specific MRR in 1999 was 1.26 (interquartile range [IQR], 1.17–1.34; range, 1.03–1.56), indicating predominance of heart disease mortality nationwide. Median state-specific MRR decreased annually through 2010, reaching a low of 1.00 (IQR, 0.95–1.07; range, 0.71–1.25), indicating that predominance of heart disease mortality prevailed in approximately half of states. Median state-specific MRR increased to 1.03 (IQR, 0.97–1.12; range, 0.77–1.31) in 2016. In 2016, age-standardized cancer mortality exceeded that for heart disease in 19 states. State-level transitions were most apparent for people aged 65 to 84 and affected men, women, and all racial/ethnic groups.

**Conclusion:**

State-level data indicated heterogeneity across US states in the predominance of heart disease mortality relative to cancer mortality. Timing and magnitude of transitions toward cancer mortality predominance varied by state.

## Introduction

In the early 1900s, the United States went through an epidemiologic transition in which chronic diseases displaced acute infections as the leading causes of death. Heart disease was the leading cause of death nationally from 1910 through 2016, except from 1918 through 1920 (1,2). Cancer was the second leading cause of death nationally from 1933 through 2016, except in 1936 and 1937 (1,2). In the mid-1900s, heart disease mortality far exceeded cancer mortality (1–3). For example, in 1963 heart disease deaths were 375.5 per 100,000 population, accounting for 39% of all deaths, and cancer deaths were 151.4 per 100,000 population, accounting for 16% of all deaths (1).

Now the United States is going through another epidemiologic transition, in which cancer may eventually become the leading cause of death nationally (2–4). Since 1964, heart disease mortality rates have steadily declined (1–6). Cancer mortality rates increased through 1991 and then started to decline, though at a much slower pace than the decline in heart disease mortality. For example, from 1991 through 2014 the cancer mortality rate decreased 25%, whereas the heart disease mortality rate decreased 47% (2). From 2012 through 2014, 23.5% of deaths were attributed to heart disease and 22.7% of deaths were attributed to cancer (2,4).

Although heart disease was the leading cause of death nationally as of 2016 (2–4), it is not so in many US states. Cancer mortality counts exceeded heart disease mortality counts in 23 US states in 2010, 21 states in 2012, and 22 states in 2014, up from only 1 state in 1993 and 2 states in 2000 (4,7,8). The objective of our study was to provide an in-depth analysis of age-standardized annual state-specific mortality rates for heart disease and cancer. We hypothesized that the timing and magnitude of transitions toward cancer mortality predominance would vary by state, and we sought to investigate this heterogeneity.

## Methods

We studied longitudinal state-specific mortality patterns in the United States by using population-based annual mortality data, which are publicly available via the National Vital Statistics System and the Centers for Disease Control and Prevention’s online integrated information and communication system, WONDER (Wide-ranging Online Data for Epidemiologic Research) (2). We obtained underlying cause-of-death data for each of the 50 states for each calendar year from 1999 through 2016 (2). Underlying cause of death was based on the *International Classification of Diseases, 10th Revision* (ICD-10) (9). We defined heart disease as ICD-10 codes I00–I09, I11, I13, and I20–I51 (diseases of heart) and cancer as ICD-10 codes C00–C97 (malignant neoplasms).

We compared age-standardized annual state-specific mortality rates for heart disease and cancer by using mortality rate ratios (MRRs), calculated as heart disease mortality rate divided by cancer mortality rate. We then made categories of MRRs (<0.7, 0.7 to <0.8, 0.8 to <0.9, 0.9 to <1.0, >1.0 to <1.1, 1.1 to <1.2, 1.2 to <1.3, 1.3 to <1.4, and ≥1.4).

Data on age-standardized rates were based mostly on 10-year age categories (<1 y, 1–4 y, 5–14 y, 15–24 y . . . 75–84 y, ≥85 y) and the year 2000 standard US population (2,10). MRRs greater than 1 indicate predominance of heart disease mortality, with the age-standardized heart disease mortality rate exceeding the age-standardized cancer mortality rate. MRRs less than 1 indicate predominance of cancer mortality, with the age-standardized cancer mortality rate exceeding the age-standardized heart disease mortality rate. MRRs equal to 1 indicate equal age-standardized heart disease and cancer mortality rates. The MRR provides richer information than ranking causes of death as first or second, because the MRR shows the magnitude of the gap between age-standardized rates of deaths from different causes.

We used age-standardized annual state-specific MRRs to generate a choropleth map and summary statistics for each calendar year, including the following values: minimum, maximum, median, and 10th, 25th, 75th, and 90th percentiles of MRR, and number of states in each MRR category. We further analyzed MRRs for selected age groups (<65 y, 65–84 y, and ≥85 y), sex (male and female), and race/ethnicity (non-Hispanic white, non-Hispanic black, Hispanic, and non-Hispanic other [consisting of American Indian/Alaska Native and Asian/Pacific Islander]). In some states, data were sparse for certain racial/ethnic groups. Therefore, we combined the first 3 calendar years of data (1999–2001) and the last 3 calendar years of data (2014–2016) to calculate stable state-specific MRRs for racial/ethnic groups. In those analyses, we considered states where heart disease or cancer mortality counts in racial/ethnic groups were less than 20 deaths per 3-year period to have insufficient data, and we did not calculate MRRs for those subgroups in those states. Although we did not round MRR calculations during our analysis, tabulated results were rounded to 2 decimal places.

Data were analyzed by using SAS version 9.4 (SAS Institute), Microsoft Excel 2016 (Microsoft Corporation), ArcMap version 10.4 (Esri), and R 3.4.3 (The R Foundation).

Age-standardized mortality rates were used to calculate MRRs because the relationship between age and heart disease mortality differs from the relationship between age and cancer mortality (3,11), and age distributions differ across states, calendar years, and demographic subgroups. State-specific MRRs and comparisons of MRRs across states, calendar years, and demographic groups may thus be confounded by age. Age-standardization reduces the effect of different age distributions and rules out age differences as an explanation for heterogeneity across states in the timing of the transition toward cancer mortality predominance.

In secondary analyses, actual mortality counts were used rather than age-standardized mortality rates to calculate unadjusted mortality count ratios (MCRs). The MCR compares the absolute number of heart disease deaths with the absolute number of cancer deaths. Comparisons of MCRs across states or calendar years may be influenced by state-specific age distributions or population aging over time. When we analyzed unadjusted MCRs, all patterns observed in our age-standardized analysis were still evident, with only minor differences. Therefore, the conclusions drawn in this report would not change, so we did not report results from the unadjusted analyses.

## Results

**Figure 1:**
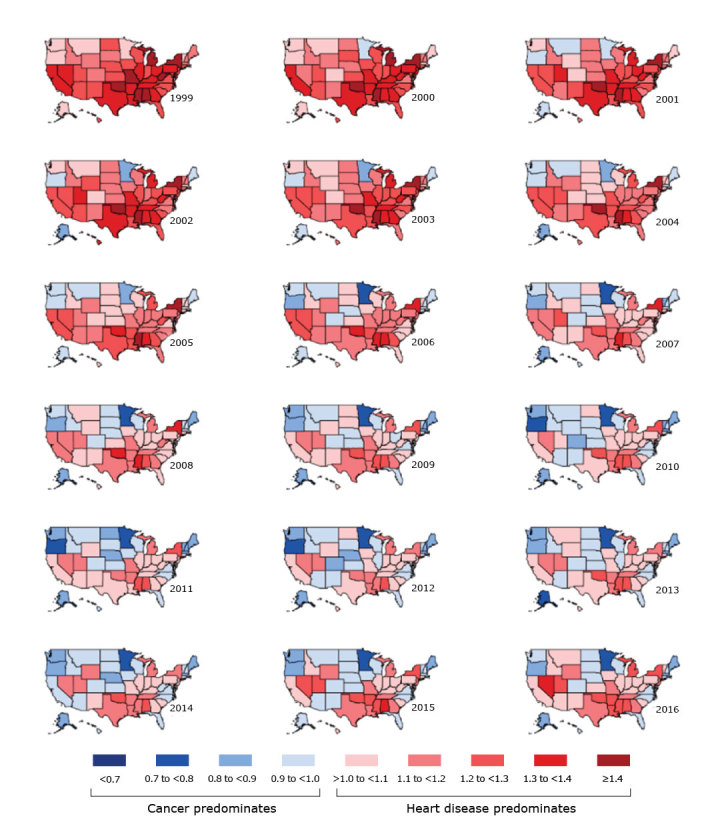
Distribution, by state, of age-standardized state-specific mortality rate ratios (MRRs), calculated by dividing the mortality rate of heart disease by the mortality rate of cancer, among all ages, both sexes, and all races and ethnicities, United States, 1999–2016. A ratio of <1.0 indicates that cancer predominates, and a ratio of >1.0 indicates that heart disease predominates.

**Figure 2:**
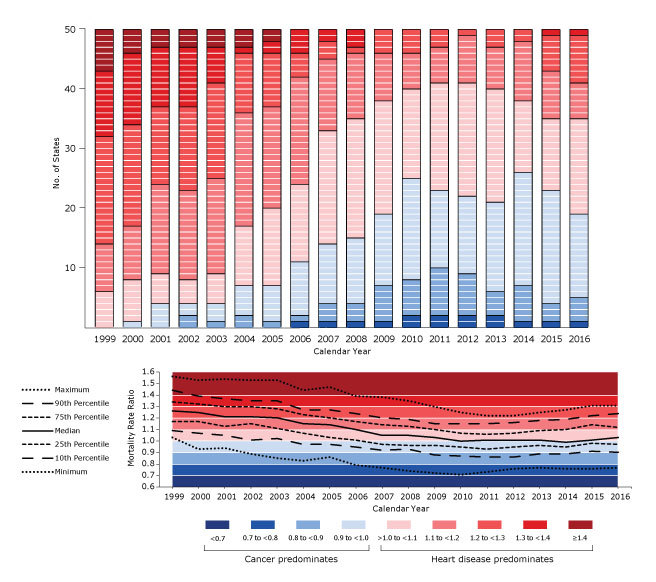
Frequency of categories of state-specific mortality rate ratios (MRRs), calculated by dividing the mortality rate of heart disease by the mortality rate for cancer, among all ages, both sexes, and all races and ethnicities, United States, 1999–2016. Top, number of states in each category of MRR, by calendar year. Each cell represents 1 state. Bottom, summary statistics by calendar year.

In 1999, age-standardized heart disease mortality exceeded that for cancer in all 50 states (Figure 1). New York State had the highest state-specific MRR (1.56), meaning that heart disease mortality was 56% higher than cancer mortality. New York State was followed by Oklahoma (1.53), Mississippi (1.52), West Virginia (1.45), Missouri (1.45), Alabama (1.44), and Michigan (1.43). Minnesota had the weakest predominance of heart disease mortality in 1999, with a MRR of 1.03, meaning that heart disease mortality was 3% higher than cancer mortality. Minnesota was followed by Oregon (1.04), Alaska (1.04), Vermont (1.07), Washington (1.09), and Montana (1.10). Among the 50 states, median state-specific MRR in 1999 was 1.26 (interquartile range [IQR], 1.17–1.34) (Figure 2).

**Table:** 

State	Age-Standardized Mortality Rate Ratio by Calendar Year^a^																	
	‘99	‘00	‘01	‘02	‘03	‘04	‘05	‘06	‘07	‘08	‘09	‘10	‘11	‘12	‘13	‘14	‘15	‘16
Alabama	1.44	1.39	1.37	1.40	1.39	1.36	1.34	1.32	1.23	1.23	1.21	1.23	1.22	1.22	1.25	1.26	1.31	1.28
Alaska	1.04	1.02	0.98	0.89	0.98	0.87	0.97	0.96	0.84	0.83	0.87	0.86	0.85	0.82	0.78	0.89	0.96	0.89
Arizona	1.24	1.20	1.19	1.20	1.17	1.13	1.16	1.10	1.04	1.06	1.02	0.95	1.01	0.98	0.96	0.96	0.98	1.02
Arkansas	1.34	1.35	1.35	1.33	1.28	1.21	1.20	1.22	1.14	1.17	1.14	1.14	1.12	1.16	1.13	1.19	1.20	1.25
California	1.39	1.32	1.30	1.30	1.29	1.24	1.20	1.21	1.12	1.11	1.05	1.03	1.05	1.02	1.03	0.99	1.02	1.02
Colorado	1.15	1.09	1.07	1.05	1.06	1.03	1.03	0.97	0.95	0.94	0.90	0.89	0.90	0.89	0.90	0.96	0.96	0.95
Connecticut	1.25	1.23	1.16	1.18	1.11	1.04	1.02	1.00	1.00	1.01	0.97	0.95	0.98	1.02	1.00	0.99	1.01	1.00
Delaware	1.21	1.29	1.18	1.23	1.23	1.14	1.17	1.08	1.06	0.95	1.02	0.95	0.97	0.95	1.00	1.01	1.00	0.96
Florida	1.30	1.25	1.26	1.23	1.20	1.16	1.12	1.08	1.04	1.01	0.98	0.98	0.95	0.96	0.96	0.99	0.99	1.00
Georgia	1.40	1.34	1.34	1.33	1.30	1.23	1.24	1.21	1.14	1.14	1.13	1.10	1.07	1.05	1.07	1.09	1.11	1.12
Hawaii	1.29	1.25	1.16	1.30	1.16	1.17	1.06	1.02	0.99	1.04	1.04	0.96	0.96	1.00	1.03	0.98	1.00	0.99
Idaho	1.18	1.19	1.20	1.19	1.11	1.11	1.05	1.05	1.01	0.94	0.98	1.00	0.99	0.99	0.93	0.98	1.02	1.06
Illinois	1.33	1.24	1.24	1.23	1.21	1.15	1.15	1.11	1.05	1.05	1.01	1.02	1.02	0.98	0.99	1.00	1.02	1.01
Indiana	1.30	1.27	1.23	1.20	1.20	1.17	1.14	1.12	1.07	1.04	1.03	1.02	1.01	1.02	1.04	1.02	1.03	1.05
Iowa	1.27	1.23	1.19	1.17	1.12	1.07	1.07	1.05	1.00	1.05	1.03	1.01	0.96	1.01	1.00	0.95	0.98	1.02
Kansas	1.23	1.25	1.16	1.18	1.15	1.09	1.04	1.03	1.01	1.01	0.95	0.96	0.93	0.94	0.96	0.94	0.96	1.00
Kentucky	1.39	1.33	1.29	1.28	1.25	1.18	1.17	1.14	1.06	1.09	1.05	1.01	1.05	1.03	1.02	1.01	1.01	1.05
Louisiana	1.31	1.27	1.24	1.22	1.25	1.19	1.23	1.17	1.18	1.16	1.16	1.16	1.10	1.12	1.14	1.16	1.18	1.24
Maine	1.11	1.09	1.06	0.98	0.99	0.94	0.91	0.90	0.91	0.89	0.84	0.80	0.82	0.81	0.87	0.87	0.88	0.88
Maryland	1.24	1.25	1.24	1.20	1.22	1.15	1.14	1.11	1.13	1.08	1.08	1.06	1.03	1.05	1.06	1.04	1.09	1.05
Massachusetts	1.11	1.05	1.06	1.02	1.04	0.99	0.96	0.92	0.93	0.93	0.89	0.88	0.86	0.85	0.89	0.88	0.91	0.90
Michigan	1.43	1.40	1.38	1.35	1.33	1.27	1.25	1.20	1.19	1.20	1.13	1.12	1.13	1.13	1.17	1.15	1.18	1.21
Minnesota	1.03	0.93	0.94	0.90	0.85	0.83	0.86	0.79	0.77	0.74	0.72	0.71	0.73	0.76	0.77	0.76	0.76	0.77
Mississippi	1.52	1.53	1.54	1.52	1.49	1.42	1.47	1.34	1.38	1.35	1.27	1.25	1.22	1.15	1.22	1.19	1.28	1.24
Missouri	1.45	1.39	1.32	1.34	1.31	1.24	1.20	1.17	1.15	1.16	1.11	1.09	1.10	1.06	1.09	1.10	1.14	1.15
Montana	1.10	1.06	1.00	1.01	1.06	0.97	0.93	0.95	0.96	1.06	0.95	0.96	0.95	0.97	1.00	0.95	0.99	1.06
Nebraska	1.24	1.17	1.14	1.16	1.11	1.08	1.02	0.95	0.95	0.96	0.93	0.92	0.90	0.89	0.92	0.90	0.98	0.91
Nevada	1.33	1.20	1.26	1.23	1.22	1.25	1.29	1.27	1.14	1.13	1.11	1.13	1.15	1.18	1.18	1.20	1.28	1.31
New Hampshire	1.16	1.15	1.20	1.11	1.12	1.04	1.00	0.98	0.96	0.94	0.89	0.91	0.85	0.87	0.94	0.92	0.92	0.92
New Jersey	1.29	1.31	1.24	1.25	1.22	1.19	1.18	1.11	1.07	1.09	1.06	1.07	1.05	1.07	1.09	1.07	1.11	1.10
New Mexico	1.26	1.14	1.22	1.13	1.15	1.13	1.14	1.13	1.06	1.01	1.03	0.99	1.02	1.00	1.01	1.01	0.99	1.08
New York	1.56	1.53	1.51	1.53	1.50	1.43	1.43	1.39	1.36	1.35	1.30	1.23	1.21	1.17	1.19	1.17	1.22	1.21
North Carolina	1.26	1.30	1.21	1.19	1.20	1.11	1.11	1.03	1.03	1.03	1.02	0.98	0.96	0.98	0.99	0.94	0.99	0.96
North Dakota	1.25	1.16	1.12	1.16	1.13	1.07	1.06	1.01	1.02	0.94	1.06	1.01	0.90	1.01	0.99	0.98	0.93	0.99
Ohio	1.33	1.32	1.31	1.25	1.22	1.17	1.17	1.11	1.05	1.08	1.00	1.02	1.03	1.03	1.06	1.05	1.09	1.07
Oklahoma	1.53	1.52	1.46	1.51	1.53	1.44	1.38	1.33	1.27	1.30	1.23	1.23	1.20	1.16	1.23	1.27	1.27	1.28
Oregon	1.04	1.00	0.98	0.98	0.96	0.91	0.91	0.89	0.88	0.85	0.82	0.79	0.78	0.78	0.83	0.82	0.85	0.87
Pennsylvania	1.34	1.31	1.28	1.26	1.23	1.19	1.17	1.10	1.08	1.09	1.06	1.03	1.04	1.01	1.05	1.04	1.06	1.07
Rhode Island	1.16	1.21	1.21	1.22	1.21	1.15	1.21	1.11	1.14	1.09	0.99	0.94	1.00	1.01	0.94	0.96	0.98	0.96
South Carolina	1.29	1.25	1.19	1.21	1.17	1.15	1.13	1.06	1.06	1.02	1.04	1.03	1.01	1.01	1.03	1.06	1.07	1.04
South Dakota	1.16	1.23	1.17	1.16	1.10	1.07	1.02	1.04	0.95	0.99	1.09	0.91	0.91	0.95	0.97	0.95	0.98	0.98
Tennessee	1.40	1.34	1.32	1.34	1.31	1.24	1.19	1.16	1.13	1.16	1.10	1.11	1.09	1.09	1.10	1.12	1.15	1.11
Texas	1.38	1.34	1.34	1.33	1.30	1.25	1.23	1.17	1.16	1.13	1.12	1.09	1.06	1.07	1.09	1.11	1.15	1.13
Utah	1.22	1.28	1.30	1.31	1.30	1.26	1.19	1.17	1.21	1.19	1.14	1.07	1.15	1.11	1.17	1.18	1.22	1.23
Vermont	1.07	1.16	1.14	1.13	1.11	1.07	1.03	1.03	0.86	0.94	0.92	0.84	0.86	0.91	0.92	0.93	0.92	1.00
Virginia	1.20	1.18	1.17	1.15	1.12	1.12	1.07	1.05	1.01	1.01	0.98	0.98	0.95	0.96	0.97	0.97	0.97	0.97
Washington	1.09	1.07	1.05	1.03	1.01	0.97	1.00	0.96	0.95	0.93	0.88	0.89	0.87	0.86	0.89	0.88	0.88	0.90
West Virginia	1.45	1.36	1.36	1.35	1.36	1.24	1.22	1.18	1.14	1.17	1.08	1.07	1.05	1.09	1.01	0.99	1.00	1.05
Wisconsin	1.25	1.24	1.17	1.15	1.13	1.06	1.04	1.01	0.97	0.97	0.96	0.95	0.93	0.97	0.96	0.96	0.98	0.98
Wyoming	1.17	1.17	1.09	1.21	1.07	1.12	1.11	1.10	1.04	1.10	1.05	0.98	1.02	1.08	1.03	1.15	1.14	1.12

^a ^In this tabular version, MRRs are rounded to 2 decimal places, such that some are listed as 1.00 when really, unrounded, they are either slightly <1.0 or slightly >1.0.

**Table:** 

Category	Calendar Year																	
	1999	2000	2001	2002	2003	2004	2005	2006	2007	2008	2009	2010	2011	2012	2013	2014	2015	2016
**No. of states in each MRR category**																		
**>1.0** **(heart disease predominates)**	50	49	46	46	46	43	43	39	36	35	31	25	27	28	29	24	27	31
≥1.4	7	4	3	3	3	3	2	0	0	0	0	0	0	0	0	0	0	0
1.3 to <1.4	11	12	10	10	6	1	2	4	2	3	0	0	0	0	0	0	1	1
1.2 to <1.3	18	17	13	14	16	10	9	4	3	1	4	4	3	1	3	2	6	8
1.1 to <1.2	8	9	15	15	16	19	17	18	12	11	8	6	6	8	7	10	8	6
>1.0 to <1.1	6	7	5	4	5	10	13	13	19	20	19	15	18	19	19	12	12	16
**<1.0 (cancer predominates)**	0	1	4	4	4	7	7	11	14	15	19	25	23	22	21	26	23	19
0.9 to <1.0	0	1	4	2	3	5	6	9	10	11	12	17	13	13	15	19	19	14
0.8 to <0.9	0	0	0	2	1	2	1	1	3	3	6	6	8	7	4	6	3	4
0.7 to <0.8	0	0	0	0	0	0	0	1	1	1	1	2	2	2	2	1	1	1
<0.7	0	0	0	0	0	0	0	0	0	0	0	0	0	0	0	0	0	0
**MRR summary statistic**																		
Maximum	1.56	1.53	1.54	1.53	1.53	1.44	1.47	1.39	1.38	1.35	1.30	1.25	1.22	1.22	1.25	1.27	1.31	1.31
90th Percentile	1.44	1.39	1.37	1.35	1.35	1.27	1.27	1.24	1.20	1.19	1.15	1.15	1.15	1.16	1.18	1.19	1.22	1.24
75th Percentile	1.34	1.32	1.30	1.30	1.28	1.23	1.20	1.17	1.14	1.13	1.10	1.07	1.06	1.07	1.09	1.10	1.14	1.12
Median	1.26	1.25	1.21	1.21	1.20	1.15	1.14	1.10	1.05	1.05	1.03	1.00	1.01	1.01	1.01	0.99	1.01	1.03
25th Percentile	1.17	1.17	1.13	1.15	1.11	1.07	1.03	1.01	0.97	0.96	0.96	0.95	0.93	0.95	0.96	0.95	0.98	0.97
10th Percentile	1.09	1.07	1.05	1.01	1.02	0.97	0.97	0.95	0.92	0.93	0.88	0.87	0.86	0.86	0.89	0.89	0.91	0.90
Minimum	1.03	0.93	0.94	0.89	0.85	0.83	0.86	0.79	0.77	0.74	0.72	0.71	0.73	0.76	0.77	0.76	0.76	0.77

From 1999 through 2016, age-standardized cancer mortality exceeded heart disease mortality (MRR <1.00) in several calendar years in 27 states (Figure 1). Higher cancer mortality than heart disease mortality occurred for at least 10 calendar years in 11 states, 5 to 9 calendar years in 13 states, and 2 to 4 calendar years in 3 states. Some states, such as Minnesota and Maine, after first transitioning from heart disease mortality predominance to cancer mortality predominance, remained consistently cancer mortality predominant every calendar year thereafter. In contrast, other states, such as Montana and Connecticut, fluctuated between cancer and heart disease mortality predominance over time. In addition to the 27 states in which cancer mortality predominated in several calendar years, 4 states were cancer mortality predominant during a single calendar year (Ohio in 2009, Wyoming in 2010, and California and West Virginia in 2014). In 19 states, age-standardized heart disease mortality exceeded cancer mortality in every calendar year studied. The transition toward state-specific cancer mortality predominance occurred steadily from 1999 through 2010, then leveled and even reversed slightly from 2011 through 2016 (Figure 2). This pattern is reflected in the median state-specific MRR over time, which was 1.26 (IQR, 1.17–1.34) in 1999, 1.00 (IQR, 0.95–1.07) in 2010, and 1.03 (IQR, 0.97–1.12) in 2016.

In 2016, age-standardized cancer mortality exceeded heart disease mortality in 19 states (Figure 1 and Figure 2). Minnesota had the strongest predominance of cancer mortality in 2016, with a MRR of 0.77, meaning that heart disease mortality was 23% lower than cancer mortality. Other states with relatively strong predominance of cancer mortality in 2016 were Oregon (0.87), Maine (0.88), Alaska (0.89), and Massachusetts (0.90). Nevada had the strongest predominance of heart disease mortality in 2016, with a MRR of 1.31, meaning that heart disease mortality was 31% higher than cancer mortality. Nevada was followed by Oklahoma (1.28), Alabama (1.28), Arkansas (1.25), Mississippi (1.24), Louisiana (1.24), Utah (1.23), New York State (1.21), and Michigan (1.21) (Figure 2).

State-level transitions from heart disease to cancer as the leading cause of death from 1999 to 2016 were most apparent in the group aged 65 to 84, for both men and women (Table 1). Among men aged 65 to 84, the number of states with cancer mortality predominance increased from 8 states in 1999 to 42 states in 2016. Similarly, among women aged 65 to 84, the number of states with cancer mortality predominance increased from 16 states in 1999 to 47 states in 2016. In contrast, men and women aged 85 or older were more likely to die of heart disease than of cancer in every state in both 1999 and 2016; MRRs in both years were 1.40 or more. For men younger than 65, the cancer mortality rate exceeded the heart disease mortality rate in 33 states in 1999 and in 27 states in 2016. For women younger than 65, the cancer mortality rate exceeded the heart disease mortality rate in every state in both 1999 and 2016; MRRs were less than <0.70 in nearly every state. For both sexes younger than 65, several states shifted toward weaker cancer mortality predominance, opposite of the trend seen for older ages.

**Table 1:** Summary Statistics for Age-Standardized State-Specific MRRs (Heart Disease Mortality Rate to Cancer Mortality Rate) for All Races and Ethnicities Groups, by Sex, Age, and Calendar Year, United States,1999–2016^a^

Category	MRR >1.0 (Heart Disease Predominant), No. of States in Each MRR Category						MRR <1.0 (Cancer Predominant), No. of States in Each MRR Category					Distribution of MRRs Across States, Percentile		
	≥1.4	1.3 to <1.4	1.2 to <1.3	1.1 to <1.2	>1.0 to <1.1	Total	0.9 to <1.0	0.8 to <0.9	0.7 to <0.8	<0.7	Total	25th	50th	75th
**Both Sexes**														
**All ages**														
1999	7	11	18	8	6	50	0	0	0	0	0	1.17	1.26	1.34
2016	0	1	8	6	16	31	14	4	1	0	19	0.97	1.03	1.12
**Aged ≥85**														
1999	50	0	0	0	0	50	0	0	0	0	0	2.88	3.14	3.46
2016	50	0	0	0	0	50	0	0	0	0	0	2.15	2.37	2.56
**Aged 65–84**														
1999	0	2	8	11	16	37	10	3	0	0	13	1.00	1.08	1.18
2016	0	0	0	0	6	6	4	15	19	6	44	0.75	0.80	0.88
**Aged <65**														
1999	0	0	0	0	0	0	3	11	10	26	50	0.62	0.70	0.81
2016	0	0	0	0	3	3	5	7	13	22	47	0.66	0.72	0.86
**Men**														
**All ages **														
1999	4	15	17	10	4	50	0	0	0	0	0	1.18	1.27	1.35
2016	1	0	11	12	19	43	6	1	0	0	7	1.02	1.10	1.19
**Aged ≥85**														
1999	50	0	0	0	0	50	0	0	0	0	0	2.07	2.32	2.42
2016	50	0	0	0	0	50	0	0	0	0	0	1.77	1.96	2.14
**Aged 65–84**														
1999	0	1	6	17	18	42	8	0	0	0	8	1.05	1.09	1.17
2016	0	0	1	0	7	8	9	26	6	1	42	0.83	0.88	0.94
**Aged <65**														
1999	0	0	1	6	10	17	15	15	3	0	33	0.87	0.96	1.05
2016	1	0	3	13	6	23	14	8	4	1	27	0.90	0.99	1.13
**Women**														
**All ages**														
1999	8	8	11	11	9	47	9	0	0	0	3	1.10	1.23	1.37
2016	0	0	4	4	5	13	19	13	4	1	37	0.87	0.92	1.05
**Aged ≥85**														
1999	50	0	0	0	0	50	0	0	0	0	0	3.53	3.82	4.27
2016	50	0	0	0	0	50	0	0	0	0	0	2.49	2.64	2.97
**Aged 65–84**														
1999	1	5	5	8	15	34	7	7	2	0	16	0.95	1.05	1.18
2016	0	0	0	0	3	3	2	9	16	20	47	0.66	0.72	0.84
**Aged <65**														
1999	0	0	0	0	0	0	0	0	1	49	50	0.36	0.45	0.52
2016	0	0	0	0	0	0	0	0	5	45	50	0.37	0.43	0.55

Abbreviation: MRR, mortality rate ratio.

a Data source: Centers for Disease Control and Prevention (2).

Some patterns were consistent across all racial/ethnic groups: strong predominance of heart disease mortality among people aged 85 or older, especially women; numerous state-specific transitions from heart disease predominance to cancer predominance in the group aged 65 to 84; and the predominance of cancer mortality among people younger than 65, especially women (Table 2). Heart disease mortality predominated more strongly on average among non-Hispanic black people than among non-Hispanic white people during 1999–2001 (median MRR, 1.26 vs 1.23) and during 2014–2016 (median MRR, 1.07 vs 1.00). Heart disease mortality also predominated more strongly on average among Hispanic people than among non-Hispanic white people during 1999–2001 (median MRR, 1.25 vs 1.23), but during 2014–2016 predominance shifted among Hispanic people more toward cancer mortality (median MRR, 0.94 vs 1.00). Cancer mortality predominated more strongly among non-Hispanic other races than among non-Hispanic white people during 2014–2016 (median MRR, 0.91 vs 1.00). Compared with the range of MRRs among non-Hispanic white people, the range among other racial/ethnic groups was broader across states.

**Table 2:** Summary Statistics for Age-Standardized State-Specific Mortality Rate Ratios (Heart Disease Mortality Rate to Cancer Mortality Rate), by Sex, Age, Race/Ethnicity, and Calendar Year, United States, 1999–2016^a^

Category	Both Sexes					Male					Female				
	No. of States With Data^b^	No. of States With MRR <1.0	Distribution of MRRs, Percentile			No. of States With Data^b^	No. of States With MRR <1.0	Distribution of MRRs, Percentile			No. of States With Data^b^	No. of States With MRR <1.0	Distribution of MRRs, Percentile		
			25th	50th	75th			25th	50th	75th			25th	50th	75th
**All Ages**															
**Non-Hispanic white**															
1999–2001	50	1	1.16	1.23	1.32	50	0	1.20	1.25	1.32	50	5	1.08	1.17	1.29
2014–2016	50	25	0.95	1.00	1.12	50	6	1.02	1.07	1.18	50	36	0.85	0.90	1.03
**Non-Hispanic black**															
1999–2001	42	2	1.16	1.26	1.36	42	8	1.04	1.12	1.19	39	1	1.23	1.35	1.50
2014–2016	44	15	0.97	1.07	1.15	44	14	0.97	1.09	1.18	42	18	0.93	1.00	1.11
**Hispanic**															
1999–2001	44	3	1.15	1.25	1.43	40	4	1.09	1.21	1.42	40	7	1.10	1.30	1.49
2014–2016	48	32	0.87	0.94	1.04	44	26	0.89	0.97	1.14	41	29	0.77	0.90	1.01
**Non-Hispanic other^c^**															
1999–2001	48	8	1.05	1.23	1.37	46	7	1.07	1.24	1.42	45	10	1.00	1.19	1.32
2014–2016	49	36	0.83	0.91	1.00	47	21	0.91	1.02	1.12	47	42	0.71	0.81	0.95
**Aged ≥85**															
**Non-Hispanic white**															
1999–2001	50	0	2.87	3.15	3.41	50	0	2.11	2.32	2.47	50	0	3.44	3.83	4.12
2014–2016	50	0	2.23	2.39	2.57	50	0	1.86	2.01	2.13	50	0	2.62	2.74	2.98
**Non-Hispanic black**															
1999–2001	37	0	2.25	2.52	2.78	31	0	1.48	1.59	1.86	33	0	2.97	3.20	3.69
2014–2016	38	1	1.79	1.97	2.13	34	1	1.33	1.49	1.65	35	1	2.14	2.42	2.66
**Hispanic**															
1999–2001	20	0	2.31	2.90	3.29	11	0	1.95	2.25	2.68	14	0	2.38	3.53	4.08
2014–2016	25	1	1.74	2.05	2.27	26	1	1.50	1.73	1.95	24	0	2.07	2.37	2.58
**Non-Hispanic other^c^**															
1999–2001	21	0	1.86	2.48	3.09	12	0	1.70	2.11	2.44	12	0	3.02	3.16	3.85
2014–2016	33	1	1.64	1.95	2.19	25	0	1.57	1.73	1.92	27	0	1.72	2.16	2.53
**Aged 65–84**															
**Non-Hispanic white**															
1999–2001	50	19	0.95	1.02	1.12	50	13	0.99	1.07	1.13	50	31	0.88	0.96	1.11
2014–2016	50	46	0.73	0.77	0.88	50	44	0.80	0.84	0.92	50	50	0.63	0.67	0.79
**Non-Hispanic black**															
1999–2001	40	8	1.02	1.12	1.23	39	24	0.86	0.97	1.04	39	2	1.19	1.30	1.49
2014–2016	42	31	0.81	0.92	1.01	39	29	0.86	0.93	1.03	39	28	0.81	0.90	1.02
**Hispanic**															
1999–2001	38	6	1.03	1.17	1.28	34	9	1.00	1.13	1.27	31	7	1.10	1.25	1.51
2014–2016	43	40	0.71	0.83	0.94	39	30	0.76	0.86	1.00	38	34	0.65	0.78	0.89
**Non-Hispanic other^c^**															
1999–2001	46	14	0.97	1.10	1.26	34	11	0.94	1.11	1.30	35	14	0.82	1.10	1.21
2014–2016	47	44	0.67	0.75	0.87	42	34	0.71	0.80	0.93	41	37	0.62	0.74	0.84
**Aged <65**															
**Non-Hispanic white**															
1999–2001	50	50	0.62	0.67	0.74	50	39	0.87	0.93	1.00	50	50	0.34	0.38	0.44
2014–2016	50	49	0.63	0.66	0.79	50	35	0.85	0.92	1.02	50	50	0.35	0.40	0.54
**Non-Hispanic black**															
1999–2001	40	29	0.90	0.94	1.01	39	6	1.05	1.11	1.20	38	37	0.69	0.77	0.86
2014–2016	42	20	0.87	1.01	1.06	39	4	1.11	1.25	1.32	38	36	0.61	0.72	0.81
**Hispanic**															
1999–2001	37	35	0.61	0.69	0.81	33	18	0.86	0.98	1.07	21	21	0.40	0.47	0.56
2014–2016	42	40	0.56	0.62	0.75	39	28	0.79	0.87	1.01	31	31	0.30	0.36	0.45
**Non-Hispanic other^c^**															
1999–2001	41	37	0.54	0.62	0.85	32	21	0.72	0.81	1.05	26	25	0.28	0.36	0.53
2014–2016	45	38	0.48	0.59	0.79	43	28	0.73	0.83	1.17	31	31	0.23	0.32	0.44

Abbreviations: MRR, mortality rate ratio.

a Data source: Centers for Disease Control and Prevention (2).

b States where heart disease or cancer mortality counts for a race/ethnicity-sex-age subgroup were <20 deaths per 3-year period were considered to have insufficient data, and MRRs were not calculated for those subgroups in those states. Years were combined to provide stable state-specific MRRs for racial/ethnic groups.

c Non-Hispanic other consisted of American Indian/Alaska Native and Asian/Pacific Islander.

## Discussion

The transition from heart disease to cancer as the leading cause of death has occurred in many US states. Our analysis, based on age-standardized annual state-specific heart disease and cancer mortality rates, showed that from 1999 through 2016, the state-specific cancer mortality rate exceeded that for heart disease in more than half of US states in several calendar years. The timing and magnitude of transition varied by state. State-specific transitions toward cancer as the leading cause of death occurred most rapidly through the mid- to late-2000s, tapering off in more recent years. In 2016, heart disease mortality was either lower than cancer mortality or was less than 10% higher than cancer mortality in most states, whereas in 1999, heart disease mortality had been more than 20% higher than cancer mortality in nearly all states.

Cancer mortality tends to exceed heart disease mortality at younger ages, whereas heart disease mortality tends to predominate at older ages (3). One study showed that by 2002 cancer became the leading cause of death nationally for people younger than 85, while heart disease remained the leading cause of death for people aged 85 or older (12). Further exploration of national data on age groups showed that this pattern persisted in more recent years. For example, in 2014, among people aged 40 to 79, the number of cancer deaths (405,885) was 45% higher than the number of heart disease deaths (280,773), whereas among people aged 80 or older, the number of heart disease deaths (325,040) was 85% higher than the number of cancer deaths (175,504) (13). However, our analysis based on age-standardized rates showed that state-level variability in the timing of the transition from heart disease to cancer as the leading cause of death cannot be explained by state-level and time-related differences in population age distributions.

The transition toward cancer as the leading cause of death applies to both sexes and to all racial/ethnic groups. One study showed that cancer mortality first surpassed heart disease mortality nationally among non-Hispanic Asians and Pacific Islanders in 2000 and among Hispanics in 2009 (4). Our analysis of state-level data stratified by sex and racial/ethnic groups showed geographic heterogeneity in the timing of the transition among these groups. The greater ratio of cancer to heart disease mortality among women younger than 65, compared with men younger than 65, may be due primarily to high mortality rates of female breast cancer among that age group. Breast cancer ranks second among cancer deaths among women, after lung cancer (13). For 1999 through 2001, heart disease mortality predominated in most states among people aged 65 to 84 of both sexes in all racial/ethnic groups, except non-Hispanic white women and non-Hispanic black men. The exception in non-Hispanic black men may have resulted from the relatively high incidence and mortality rates of lung and prostate cancers among black men in the United States (13). During 1999–2001, among men aged 65 to 84, the age-standardized mortality rate of lung cancer among non-Hispanic black men (557.1 per 100,000) was 25% higher than among non-Hispanic white men (444.3 per 100,000), and the age-standardized mortality rate of prostate cancer among non-Hispanic black men (383.9 per 100,000) was 167% higher than among non-Hispanic white men (143.6 per 100,000) (2). The strong predominance of heart disease mortality among non-Hispanic black women aged 65 to 84 in the Southeast during 1999–2001 is perhaps partly explained by relatively low levels of lung cancer among black women (13). During 1999–2001, among women aged 65 to 84, age-standardized lung cancer mortality was 14% lower among non-Hispanic black women (209.5 per 100,000) than among non-Hispanic white women (242.6 per 100,000) (2). These patterns remained evident during 2014–2016.

The driving force behind these transitions, as documented in previous reports, is a decline in heart disease mortality (4–8,14). Possible causes for the patterns we observed include state-level and time-related differences in tobacco smoking, other risk factors for heart disease and cancer, and successful treatment of heart disease and cancer. Smoking prevalence in the United States peaked in 1964 and began falling thereafter (15). A 20- to 30-year latency period between smoking and lung and other cancers is suggested by the fact that cancer death rates did not begin to decline until after 1991, whereas the reduction in heart disease mortality rates correlates more closely in time with the reduction in tobacco smoking (16). Tobacco smoking is strongly associated with lung cancer (17–19), but the decrease in age-standardized lung cancer mortality rate during the study period was 31% (from 55.4 per 100,000 in 1999 to 38.3 per 100,000 in 2016) as opposed to a decrease in age-standardized heart disease mortality rate of 38% (from 266.5 per 100,000 in 1999 to 165.5 per 100,000 in 2016) (2). The more rapid reduction in heart disease mortality than in lung cancer mortality suggests that other risk factors for heart disease were declining during the same time. Environmental tobacco smoke policies changed during the study period, and they differ across states and localities (20). Reductions in environmental tobacco smoke would be expected to reduce heart disease mortality more quickly, with a relative lag in reduction of cancer mortality. In addition, one study noted that improvements in the treatment of risk factors for heart disease have been greater than improvements in the early detection of cancer (21). The treatment of cardiovascular disease has also advanced in the past 5 decades (5,21). Geographic patterns of heart disease mortality in the United States and shifts in these patterns over time are detailed in recent comprehensive reports (22,23), but these reports do not refer to geographic or time-related patterns of cancer mortality. Thus our report adds value by directly comparing heart disease mortality with cancer mortality.

Although the relationship between heart disease and cancer mortality at the national level is well known, few state-specific in-depth explorations of heart disease versus cancer mortality are available. One such study, using mortality data from 2006 through 2009, described a transition toward cancer mortality predominance in Kentucky and Texas (24,25). This study observed a narrowing gap, driven by declining heart disease mortality, between heart disease mortality and cancer mortality in both states. A strength of our analysis was the ability to examine such patterns not only for selected states but for all 50 states, and for both sexes, selected age groups, and several racial/ethnic groups.

Our study has limitations. The validity of our study depends on the accuracy and completeness of death certificate data. We used data on underlying cause of death to be consistent with the National Center for Health Statistics’ methodology for ranking causes of death (26), although we recognize that comorbidities, along with the subjectivity inherent in the process of determining cause of death, may lead to inconsistencies in the coding of underlying cause of death on death certificates. Data on racial/ethnic groups were incomplete because for race/ethnicity-specific analyses we excluded death certificates that did not indicate whether the decedent was Hispanic or non-Hispanic. In addition, certain racial/ethnic groups in certain states were represented by small populations in our analysis, but we mitigated this shortcoming by aggregating data across multiple calendar years.

The oft-repeated message that “heart disease is the leading cause of death in the United States,” while true at the national level as of 2016, obscures the heterogeneity in the relative predominance of heart disease mortality or cancer mortality across states and over time. Our longitudinal analysis of state-level data enriches our understanding of this epidemiologic transition toward cancer becoming the leading cause of death in the United States. Our findings can aid public health and medical professionals in interpreting vital statistics, creating public health messaging, and setting priorities in chronic disease prevention and control programs, particularly at the state level.
